# The Potential Value of Gastric Histopathology for Predicting Colorectal Adenomatous Polyps Among the Chinese Population: A Retrospective Cross-Sectional Study

**DOI:** 10.3389/fonc.2022.889417

**Published:** 2022-07-06

**Authors:** Weiwei Li, Lin Zhang, Yuanming Jing, Yanfei Yang, Yulong Wang

**Affiliations:** ^1^ Department of Gastroenterology, Shaoxing People’s Hospital, Shaoxing, China; ^2^ Department of Clinical Pharmacy, Shaoxing People’s Hospital, Shaoxing, China; ^3^ Department of Gastrointestinal Surgery, Shaoxing People’s Hospital, Shaoxing, China; ^4^ Department of Anesthesiology, Shaoxing People’s Hospital, Shaoxing, China

**Keywords:** gastric histopathology, colorectal adenomatous polyps, colorectal cancer, retrospective cross-sectional study, logistic regression analysis

## Abstract

**Background:**

It remains unknown whether gastric histopathology is associated with the occurrence of colonic neoplasms. We aimed to clarify the association between gastric histopathology and different types of colorectal polyps (CP) and colorectal cancer (CRC), and whether various gastric histopathologies are risk factors for different types of CP and CRC.

**Methods:**

A retrospective cross-sectional study was conducted on 5,986 patients who underwent gastroscopy and colonoscopy simultaneously at Shaoxing People’s Hospital from August 1, 2019, to May 31, 2020. The Pearson χ^2^ test was used to analyze the occurrence of various gastric histopathologies in different types of CP and CRC, and logistic regression was used to determine whether various gastric histopathologies were risk factors for different types of CP and CRC.

**Results:**

For the Chinese population, male sex (odds ratio [OR] 1.67, 95% confidence interval [CI] 1.41–1.97, *P* < 0.001) and old age (OR 1.03, 95% CI 1.02–1.04, *P* < 0.001) were risk factors for non-adenomatous polyps (NAP), but *Helicobacter pylori* (*H. pylori*) and various gastric histopathologies were not significant in the NAP compared with the normal group. Nevertheless, it is noteworthy that, similar to male sex and old age, *H. pylori* (OR 1.22, 95% CI 1.08–1.38, *P =* 0.002), low-grade intraepithelial neoplasia (LGIN) (OR 1.79, 95% CI 1.21–2.66, *P =* 0.004), gastric fundus gland polyps (FGPs) (OR 1.44, 95% CI 1.11–1.87, *P =* 0.007), hyperplastic/inflammatory gastric polyps (GHP or GIP) (OR 1.50, 95% CI 1.06–2.12, *P =* 0.022), and atrophy/intestinal metaplasia (AG or IM) (OR 1.27, 95% CI 1.13–1.43, *P* < 0.001) were all risk factors for colorectal adenomatous polyps (AP). However, the results of CRC showed that old age (OR 1.13, 95% CI 1.10–1.16, *P* < 0.001) and *H. pylori* (OR 1.67, 95% CI 0.99–2.75, *P* < 0.05) were risk factors for CRC (OR 1.67, 95% CI 0.99–2.75, *P* < 0.05), but not sex and various gastric histopathologies (*P* > 0.05).

**Conclusion:**

Gastric histopathology, such as AG or IM, LGIN, FGP, and GHP or GIP, were risk factors for AP, but not for NAP and CRC, indicating that gastric histopathology has potential predictive value for AP in the Chinese population.

## Introduction

The incidence of colorectal cancer (CRC) ranks second worldwide among malignant tumors (1), which accounted for 881,000 deaths in 2018, representing 9.8% of deaths worldwide (2), and is increasing annually. Studies have confirmed that the vast majority of CRC cases evolve from colorectal polyps (CP), especially colorectal adenomatous polyps (AP) (3). Therefore, early screening for AP is important to prevent CRC occurrence.

Previous studies have shown an increased risk of colonic neoplasms in patients with upper gastrointestinal diseases, such as gastric polyps (4, 5), *H. pylori* infection (6–8), and reflux esophagitis (9), which may be related to the destruction of the gastric acid barrier (10) and long-term treatment of proton pump inhibitors (11, 12). However, whether gastric histopathology, which can better reflect the pathological state of upper gastrointestinal diseases such as gastric acid excretion and gastric acid barrier function, is related to the occurrence of colonic neoplasms has not been reported in the literature.

Therefore, this study was conducted on 5,986 patients who underwent bidirectional endoscopy (gastroscopy plus colonoscopy) to determine the association between gastric histopathology and the different types of CP and CRC and determine whether various gastric histopathologies are risk factors for different types of CP and CRC. We attempted to reveal that a decrease in gastric acid secretion and partial destruction of gastric barrier function take effect in the occurrence and progression of colonic neoplasms possibly.

## Materials and Methods

### 2.1 Clinical Data

This was a retrospective observational study. The research plan was discussed and approved by the Academic Ethics Committee of Shaoxing People’s Hospital (ethics code: 013). Patients who underwent gastroscopy and colonoscopy simultaneously in Shaoxing People’s Hospital from August 1, 2019, to May 31, 2020, were selected and enrolled. The inclusion criteria were as follows: patients who signed the informed consent form before the examination, had complete clinical data, had a good bowel preparation, and underwent a whole gastroenteroscopy examination. The exclusion criteria were as follows: partial or total gastrointestinal resection due to gastric cancer, colorectal cancer, or other causes; hereditary polyposis, including Peutz–Jeghers syndrome, and familial adenomatous polyposis; previous inflammatory bowel disease or a history of other intestinal diseases; *Helicobacter pylori* (*H. pylori*) treatment history; and no tissue biopsy during examination.

### 2.2 Research Methods

**Figure 1:**
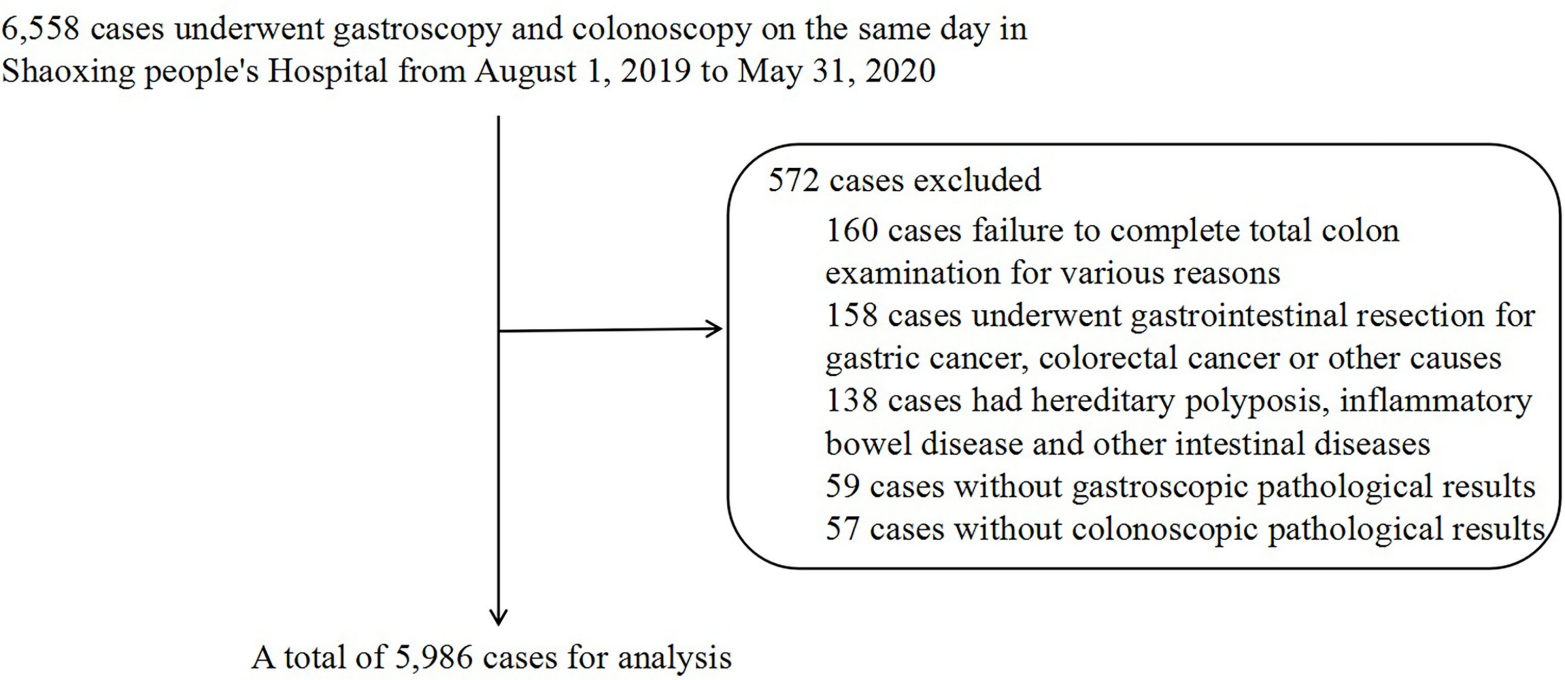
Flowchart of case selection.

Between August 1, 2019, and May 31, 2020, 6,558 cases, including inpatients, outpatients, and physical examinees, were collected through the institutional clinical information, image archive, and communication system. Finally, 5,986 cases were included for analysis (**Figure 1**). The diagnostic criteria for different types of CP and CRC were based on colonoscopy and pathological examination, and the various gastric histopathology results were obtained from the gastroscopic pathological reports. Pathological specimens were investigated by the Department of Pathology and were fixed with 10% formaldehyde solution, then embedded, sectioned, and stained with hematoxylin and eosin for histological assessment and classification. Hematoxylin and eosin (HE) staining of the gastric mucosa specimens was used to detect *H. pylori* infection.

Based on colonoscopy reports and pathological reports, the results of colonoscopy were divided into four groups: normal, non-adenomatous polyps (NAP; including proliferative polyps and inflammatory polyps), AP (including serrated adenomas, tubular adenomas, and villous adenoma), and CRC. The general information of AP, including site (distal, proximal, and multiple), number (single and multiple), and size (<1 cm and ≥1 cm), was retrospectively analyzed. Regarding the site, the right colon was defined as the proximal end, the left colon and rectum were defined as the distal end, and multiple polyps were defined as the presence of both proximal and distal polyps. HE staining of the gastric mucosa specimens was used to detect the infection status of *H. pylori*, which was divided into *H. pylori-*positive and *H. pylori-*negative. According to the gastroscopic pathology reports, the various gastric histopathologies were non-atrophic gastritis (NAG, acute gastritis, or chronic gastritis shown in pathological reports were defined as non-atrophic gastritis), atrophy/intestinal metaplasia (AG or IM), gastric fundus gland polyps (FGP), hyperplastic/inflammatory gastric polyps (GHP or GIP), adenomatous gastric polyps (GAP), low-grade intraepithelial neoplasia (LGIN), high-grade intraepithelial neoplasia (HGIN), and stomach cancer of pathology (SC-patho).

### 2.3 Statistical Methods

R language (version 4.0.4) software was used for data analysis and visualization. Specifically, the dplyr and compareGroups packages were used for data and statistical processing, ggplot2 packages were used for data visualization, and the ggpmisc, sjlabelled, and ggforest packages were used to calculate the regression equation and R-square adjustment and draw forest figures. Categorical variables were statistically represented in proportion and compared using the chi-square test or Fisher’s exact probability method. Normally distributed continuous variables were described as means ± standard deviations (`x±s) using the t-test. Enumeration data were expressed as rates or percentages, and the χ2 statistical test was used for comparison between groups. Multivariate logistic regression was used to determine whether various gastric histopathologies are risk factors for different types of CP and CRC, and the results were reported as an adjusted odds ratio (OR) with a corresponding 95% confidence interval (CI). For all analyses, *P* < 0.05 indicated a statistically significant difference.

## Results

### 3.1 The Demographic Characteristics and Occurrence of Various Gastric Histopathologies in Different Types of CP and CRC

Among the 5,986 cases, 3,707 were normal, 689 had NAP, 1,516 had AP, and 74 had CRC diagnosed by colonoscopy and biopsy. The proportion of female patients (59.24%) was higher than that of male patients (40.76%) for normal diagnoses. In contrast, in the NAP (52.54%, 47,46%), AP (55.80%, 44.20%), and CRC (54.05%, 45.95%) groups, the proportion of male patients was higher than that of female patients (*P* < 0.001). The average age in the normal group was the lowest (49.9 ± 10.90), while the patients in the CRC group (62.3 ± 10.40) were older than those in the NAP (53.3 ± 9.50) and AP (55.9 ± 9.23) groups (*P* < 0.001) (**Table 1**).

**Table 1:** The Pearson χ^2^ test and t test were used to compare the demographic characteristics and the occurrence of various gastric histopathology of cases in NAP, AP, CRC, and normal group.

	NAP *N (%)*	AP *N (%)*	CRC *N (%)*	Normal *N (%)*	*P*
Grand total	689 (100)	1,516 (100)	74 (100)	3,707 (100)	
*Demographics*					
Female	327 (47.46)	670 (44.20)	34 (45.95)	2,196 (59.24)	**<0.001**
Male	362 (52.54)	846 (55.80)	40 (54.05)	1,511 (40.76)	
Mean age (SD)	53.3 (9.50)	55.9 (9.23)	62.3 (10.40)	49.9 (10.90)	**<0.001**
*Gastric histopathology*					
NAG	343 (49.78)	719 (47.42)	36 (48.65)	2,226 (60.05)	**<0.001**
AG or IM	253 (36.72)	609 (40.17)	28 (37.84)	1,098 (29.62)	**<0.001**
*H. pylori*	186 (27.00)	454 (29.95)	24 (32.43)	989 (26.78)	0.081
FGP	83 (12.05)	197 (13.00)	8 (10.81)	364 (9.82)	**0.007**
GHP or GIP	30 (4.35)	67 (4.42)	2 (2.70)	106 (2.86)	**0.017**
GAP	1 (0.15)	7 (0.46)	2 (2.70)	4 (0.11)	**0.002**
LGIN	22 (3.19)	49 (3.23)	3 (4.05)	55 (1.48)	**<0.001**
HGIN	3 (0.44)	3 (0.20)	1 (1.35)	8 (0.22)	0.151
SC-patho	1 (0.15)	3 (0.20)	3 (4.05)	8 (0.22)	**0.002**

NAP, non-adenomatous polyps; AP, colorectal adenomatous polyps; CRC, colorectal cancer; NAG, non-atrophic gastritis; AG or IM, atrophy/intestinal metaplasia; FGP, gastric fundus gland polyp; GHP or GIP, hyperplastic/inflammatory gastric polyps; GAP, adenomatous gastric polyps; LGIN, low-grade intraepithelial neoplasia; HGIN, high-grade intraepithelial neoplasia; SC-patho, stomach cancer of pathology. P < 0.05 showed in bold.

The occurrence of various gastric histopathologies was different in the NAP, AP, and CRC groups compared to the normal group. The proportions of NAG in the normal group (60.05%) was significantly higher than that in the NAP (49.78%), AP (47.42%), and CRC (48.65%) groups (P < 0.001). The proportion of AG or IM, FGP, GAP, and LGIN in the NAP (36.72%, 12.05%, 0.15%, 3.19%), AP (40.17%, 13.00%, 0.46%, 3.23%), and CRC (37.84%, 10.81%, 2.70%, 4.05%) groups were significantly higher than that in the normal group (29.62%, 9.82%, 0.11%, 1.48%) (*P* < 0.05). The same trend was observed for *H. pylori*, although the *P*-value was 0.081. The occurrence of GHP or GIP in the NAP (4.35%) and AP (4.42%) groups was higher than that in the normal group (2.86%) but not in the CRC group (2.70%) (*P =* 0.017). However, in the CRC group, the occurrence of HGIN (1.35%) and SC-patho (4.05%) was significantly higher than in the other three groups, although the former had no statistical difference, which may be related to the small number of patients (**Table 1**).

### 3.2 Multivariate Logistic Regression Was Used to Analyze Whether Various Gastric Histopathologies Were Risk Factors for the NAP, AP, and CRC Groups Compared With the Normal Group

Data on sex, age, *H. pylori*, and various gastric histopathologies (except GAP, HGIN, and SC-patho, because of their small numbers to carry out logistic regression analysis) of 5,986 cases were included to construct the multivariate logistic regression complex model and simple model.

#### 3.2.1 Multivariate Logistic Regression for the NAP Group Compared With the Normal Group

**Figure 2:**
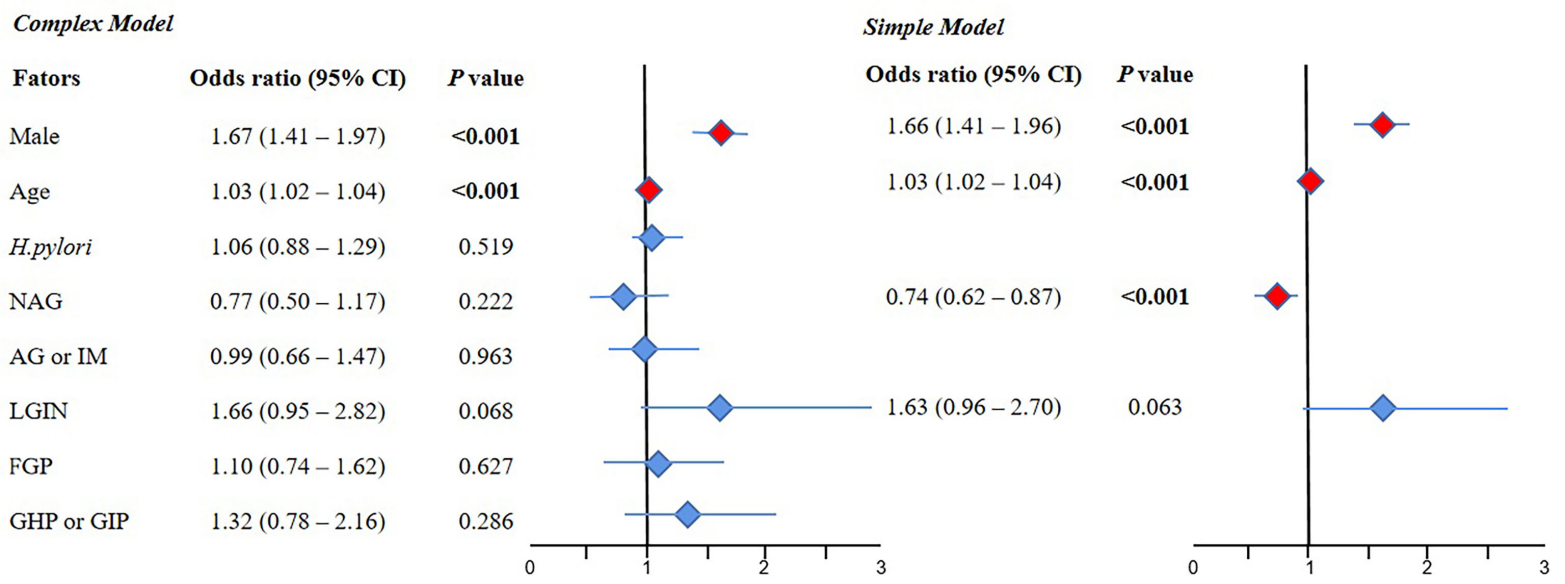
The results of multivariate logistic regression for the NAP group compared with the normal group. 95% CI, 95% confidence interval; NAG, non-atrophic gastritis; AG or IM, atrophy/intestinal metaplasia; FGP, gastric fundus gland polyp; GHP or GIP, hyperplastic/inflammatory gastric polyp; LGIN, low-grade intraepithelial neoplasia. **Complex model:** All variables were included, and then *P* values were calculated separately. **Simple model:** Based on the complex model and the stepwise regression method, an optimal simple model is obtained, which is automatically screened according to the Akaike information criterion (AIC) minimization principle.

The results of the complex model showed that male sex (OR 1.67, 95% CI 1.41–1.97, *P* < 0.001) and old age (OR 1.03, 95% CI 1.02–1.04, *P* < 0.001) were risk factors for NAP, but *H. pylori* and various gastric histopathologies were not significant. Further simple model analysis was performed and showed the same results as the complex model in sex and age; meanwhile, NAG was observed as a protective factor for NAP (OR 0.74, 95% CI 0.62–0.87, *P* < 0.001) (**Figure 2**).

#### 3.2.2 Multivariate Logistic Regression for the AP Group Compared With the Normal Group

**Figure 3:**
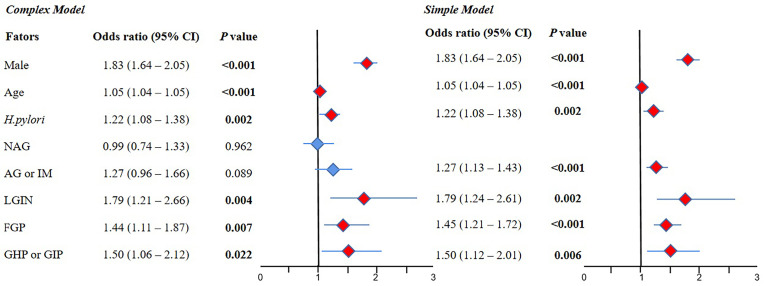
The result of multivariate logistic regression for the AP group compared with the normal group. 95% CI, 95% confidence interval; NAG, non-atrophic gastritis; AG or IM, atrophy/intestinal metaplasia; FGP, gastric fundus gland polyps; GHP or GIP, hyperplastic/inflammatory gastric polyps; LGIN, low-grade intraepithelial neoplasia. **Complex model:** All variables were included, and then *P* values were calculated separately. **Simple model:** Based on the complex model and the stepwise regression method, an optimal simple model is obtained, which is automatically screened according to the Akaike information criterion (AIC) minimization principle.

The results of the complex model showed that male sex (OR 1.83, 95% CI 1.64–2.05, *P* < 0.001), old age (OR 1.05, 95% CI 1.04–1.05, *P* < 0.001), *H. pylori* (OR 1.22, 95% CI 1.08–1.38, *P =* 0.002), LGIN (OR 1.79, 95% CI 1.21–2.66, *P =* 0.004), FGP (OR 1.44, 95% CI 1.11–1.87, *P =* 0.007), and GHP or GIP (OR 1.50, 95% CI 1.06–2.12, *P=*0.022) were risk factors for AP, but NAG and AG or IM were not significant. Further simple model analysis showed the same results as the complex model in sex, age, *H. pylori*, LGIN, FGP, and GHP or GIP, wherein AG or IM was also a risk factor for AP (OR 1.27, 95% CI 1.13–1.43, *P* < 0.001) (**Figure 3**).

#### 3.3.3 Multivariate Logistic Regression for the CRC Group Compared With the Normal Group

**Figure 4:**
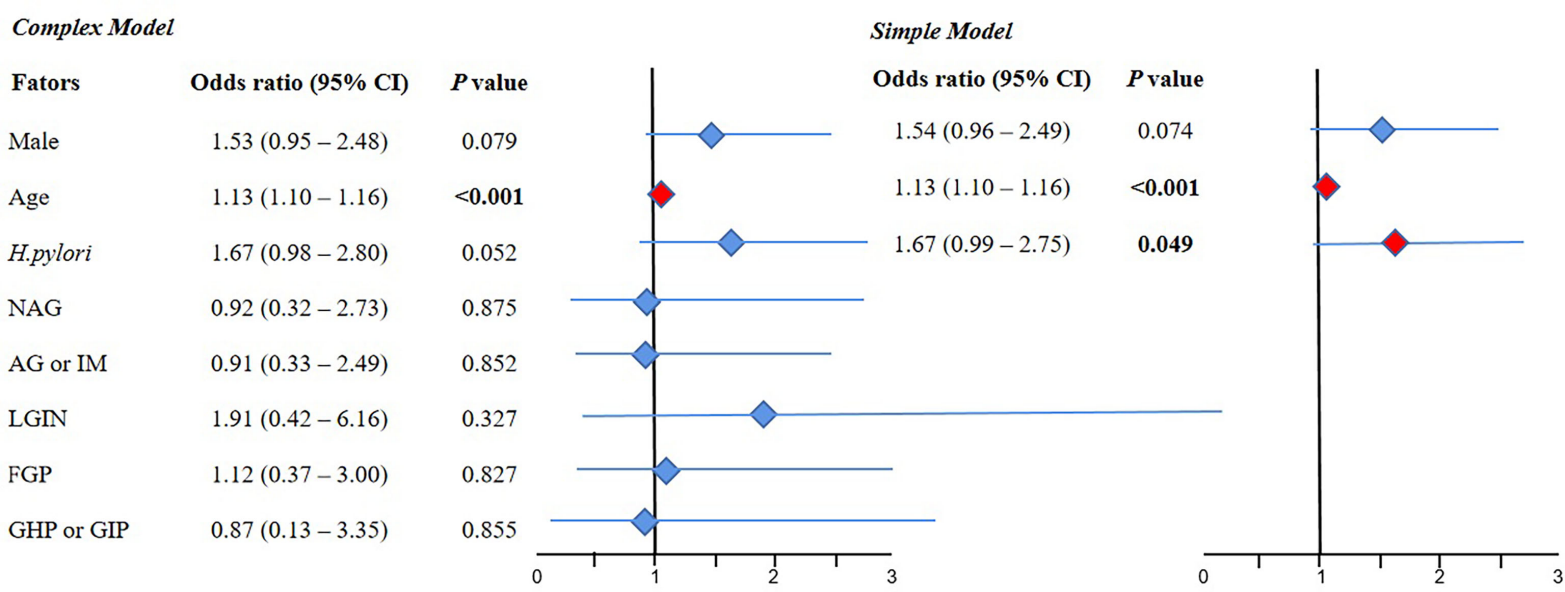
The result of multivariate logistic regression for the CRC group compared with the normal group. 95% CI, 95% confidence interval; NAG, non-atrophic gastritis; AG or IM, atrophy/intestinal metaplasia; FGP, gastric fundus gland polyps; GHP or GIP, hyperplastic/inflammatory gastric polyps; LGIN, low-grade intraepithelial neoplasia. **Complex model:** All variables were included, and then *P* values were calculated separately. **Simple model:** Based on the complex model and the stepwise regression method, an optimal simple model is obtained, which is automatically screened according to the Akaike information criterion (AIC) minimization principle.

The results of the complex model showed that old age (OR 1.13, 95% CI 1.10–1.16, *P* < 0.001) was a risk factor for CRC, but sex, *H. pylori* infection, NAG, AG or IM, LGIN, FGP, and GHP or GIP were not significant. Further simple model analysis showed the same results as the complex model in age, wherein *H. pylori* was also a risk factor for CRC (OR 1.67, 95% CI 0.99–2.75, *P* < 0.05) (**Figure 4**).

### 3.3 The Association Between Various Gastric Histopathologies and the Site, Number, and Size of AP

Among the 1,516 cases of AP, 705 cases were distal, 408 were proximal, and 403 were multiple. The proportion of multiple polyps cases was significantly higher in male patients (64.76%) than in female patients (35.24%, *P* < 0.001). The average age of multiple polyps patients was higher (58.4 ± 7.92) than that of distal (54.4 ± 9.77) and proximal (56.0 ± 8.96) polyps patients (*P* < 0.001). However, no statistical differences were observed in *H. pylori* infection and various gastric histopathologies (**Table 2**). There were 778 cases of single AP and 738 cases of multiple AP. The proportion of multiple polyps cases in male patients (62.74%) was significantly higher than that in female patients (37.26%, *P* < 0.001). The average age of the multiple group was higher (57.6 ± 8.17) than that of the single group (54.3 ± 9.88, *P* < 0.001). In addition, the proportion of NAG in the single group (51.03%) was higher than that in the multiple group (43.63%; *P =* 0.005). In contrast, the proportion of GHP or GIP and LGIN in the multiple group (5.56%, 4.34%) was higher than that in the single group (3.34%, 2.19%, *P* < 0.05) (**Table 2**). Regarding the size of AP, there were 1,149 cases with <1 cm and 367 cases with ≥1 cm. The proportion of male patients with ≥1 cm (62.67%) was significantly higher than that of female patients (37.33%, *P* = 0.003). The average age of patients with ≥1 cm was higher (57.9 ± 9.23) than that of patients with <1 cm (55.2 ± 9.14, *P* < 0.001). However, no statistical differences were observed in *H. pylori* infection and various gastric histopathologies (**Table 2**).

**Table 2:** The Pearson χ^2^ test and t test were used to compare the association between various gastric histopathologies and the site, number, and size of AP.

	Site of AP				Number of AP			Size of AP		
	Distal *N (%)*	Proximal *N (%)*	Multiple *N (%)*	*P*	Single *N (%)*	Multiple *N (%)*	*P*	<1 cm *N (%)*	≥1 cm *N (%)*	*P*
Grand total	705 (100)	408 (100)	403 (100)		778 (100)	738 (100)		1,149 (100)	367 (100)	
*Demographics*										
Female	321 (54.47)	207 (50.74)	142 (35.24)	**<0.001**	395 (50.77)	275 (37.26)	**<0.001**	533 (46.39)	137 (37.33)	**0.003**
Male	384 (45.53)	201 (49.26)	261 (64.76)		383 (49.23)	463 (62.74)		616 (53.61)	230 (62.67)	
Mean age (SD)	54.4 (9.77)	56.0 (8.96)	58.4 (7.92)	**<0.001**	54.3 (9.88)	57.6 (8.17)	**<0.001**	55.2 (9.14)	57.9 (9.23)	**<0.001**
*Gastric histopathology*										
NAG	356 (50.5)	177 (43.4)	186 (46.2)	0.061	397 (51.03)	322 (43.63)	**0.005**	546 (47.5)	173 (47.1)	0.947
AG or IM	277 (39.3)	170 (41.7)	162 (40.2)	0.738	294 (37.8)	315 (42.7)	0.059	464 (40.4)	145 (39.5)	0.813
*H. pylori*	203 (28.8)	124 (30.4)	127 (31.5)	0.620	216 (27.8)	238 (32.2)	0.064	330 (28.7)	124 (33.8)	0.075
FGP	79 (11.2)	63 (15.4)	55 (13.6)	0.116	91 (11.7)	106 (14.4)	0.142	148 (12.9)	49 (13.4)	0.885
GHP or GIP	27 (3.83)	17 (4.17)	23 (5.71)	0.329	26 (3.34)	41 (5.56)	**0.049**	56 (4.87)	11 (3.00)	0.169
GAP	2 (0.28)	2 (0.49)	3 (0.74)	0.430	1 (0.13)	6 (0.81)	0.063	4 (0.35)	3 (0.82)	0.370
LGIN	20 (2.84)	13 (3.19)	16 (3.97)	0.590	17 (2.19)	32 (4.34)	**0.026**	34 (2.96)	15 (4.09)	0.371
HGIN	2 (0.28)	1 (0.25)	0 (0.00)	0.800	3 (0.39)	0 (0.00)	0.250	2 (0.17)	1 (0.27)	0.565
SC-patho	1 (0.14)	1 (0.25)	1 (0.25)	1.000	1 (0.13)	2 (0.27)	0.615	3 (0.26)	0 (0.00)	1.000

AP, colorectal adenomatous polyps; NAG, non-atrophic gastritis; AG or IM, atrophy/intestinal metaplasia; FGP, gastric fundus gland polyp; GHP or GIP, hyperplastic/inflammatory gastric polyps; GAP, adenomatous gastric polyps; LGIN, low-grade intraepithelial neoplasia; HGIN, high-grade intraepithelial neoplasia; SC-patho, stomach cancer of pathology. P < 0.05 showed in bold.

## Discussion

The incidence and mortality of CRC are continuously increasing worldwide (13), and the majority of cases evolve from AP. Therefore, early colonoscopy screening is important to prevent the occurrence of CRC. In addition, as a digestive tract disease, some upper gastrointestinal diseases showed a potential association with colonic neoplasm (4–9), which may be related to genetic and environmental factors (14, 15), destruction of the gastric acid barrier (10), and long-term treatment of proton pump inhibitors (11, 12). The specific molecular mechanism is still unclear. Thus, we carried out a retrospective study based on 5,986 cases in China to determine the association between gastric histopathology, which can better reflect the pathological state of upper gastrointestinal diseases, and the different types of CP and CRC, to provide clues and evidence to explore the specific molecular mechanism.

Our study found that the proportions of AG or IM, FGP, GAP, and LGIN in NAP, AP, and CRC were all significantly higher than those in the normal group, and the occurrence of HGIN and SC-patho in the CRC group was significantly higher than that in the other three groups. This result was practically consistent with a previous study, which showed that patients with any type of CP (hyperplastic polyp, sessile serrated adenoma/polyp, tubular adenoma) have the significantly higher proportions of IM, FGP, and GHP compared with the control group (16). In this part of our study, the advantage was that we have included almost all types of gastric histopathology and divided colonoscopy results into four groups, which was unprecedented. Further logistic regression analysis found that *H. pylori*, AG or IM, LGIN, FGP, and GHP or GIP were all risk factors for AP, but not for NAP, wherein the results seem to be explained by the different pathogenesis of AP and NAP. Nevertheless, with the exception of *H. pylori*, various gastric histopathologies were not risk factors for CRC. Based on the fact that the majority of CRC cases evolve from colorectal adenomatous polyps, the different results between CRC and AP may be attributed to the fewer cases of CRC to conduct statistical analysis, which will be remedied by future cohort studies. In addition, further analysis of AP found that gastric histopathology has nothing to do with the site and size. The proportion of GHP or GIP and LGIN in multiple cases was higher than that in single cases, which seems to reveal that the pathological changes in the upper gastrointestinal tract have an overall impact on the lower gastrointestinal tract.

It is noteworthy that there are some potential connections and similarities between the risk factors of AP (*H. pylori*, AG or IM, LGIN, FGP, GHP, or GIP) found in our study. *H. pylori*, identified as a class I carcinogen by the World Health Organization, is recognized as the main pathogenic factor of atrophic gastritis, peptic ulcer, and gastric cancer (17–19). *H. pylori* infection leads to chronic gastritis, and long-term chronic gastritis causes atrophy of gastric mucosa and intestinal metaplasia, followed by decreased gastric acid secretion (10). Dysplasia of the gastric glands can occur due to atrophy and intestinal metaplasia, including LGIN. In addition, GHP and GIP are more likely to happen in the gastric mucosa of patients with long-term chronic gastritis or atrophy. Gastric FGP is associated with long-term oral anti-secretory medications (20, 21). The similarities of these gastric histopathologies are that they can all represent the decrease in gastric acid secretion, which will destroy the gastric acid barrier, so that microorganisms invade the lower gastrointestinal tract and then promote the occurrence and progression of AP (22). However, why the same result did not appear in NAP is not only an interesting finding of our study but also a question worthy of in-depth analysis and exploration.

The strength of our study is that various gastric histopathologies were collected and combined with endoscopic and pathological results, and CP were classified in detail according to the risk of progression to CRC, which is conducive for comparative analysis and has more guiding significance in clinical practice. Additionally, another highlight of this study is the large sample size. However, our study also had a few limitations. Firstly, this study was a single-center retrospective cross-sectional study; although the total sample size was large, there were fewer cases of CRC, which requires further large-sample cohort studies. Secondly, the duration of *H. pylori* infection was not taken into account, which was difficult to track. Thirdly, we did not include more variables for analysis, such as smoking, drinking, and eating habits, which may have impacted the final research results. In the future, prospective multicenter cohort studies with larger sample sizes should be conducted, and further investigation is required to explore specific molecular mechanisms.

In conclusion, the occurrence of various gastric histopathologies was different in NAP, AP, and CRC compared with the normal group. Gastric histopathologies, such as AG or IM, LGIN, FGP, and GHP or GIP, which can all represent the decrease in gastric acid secretion with subsequent destruction of the gastric acid barrier, were risk factors for AP, indicating that gastric histopathology has a potential predictive value for AP among the Chinese populations.

## Data Availability Statement

The raw data supporting the conclusions of this article will be made available by the authors, without undue reservation.

## Ethics Statement

The studies involving human participants were reviewed and approved by Academic Ethics Committee of Shaoxing People’s Hospital. Written informed consent to participate in this study was provided by the participants’ legal guardian/next of kin.

## Author Contributions

Concept and design: WL and YW. Acquisition, analysis, or interpretation of data: WL, YY, and YW. Drafting of the manuscript: WL. Statistical analysis: LZ and YJ. All authors contributed to the article and approved the submitted version.

## Funding

This work was supported by the Project of Zhejiang Medical and Health Science and Technology (No. 2022KY1290) and the Key Project of Shaoxing People’s Hospital Youth Scientific Research Fund (No. 2019YA02).

## Conflict of Interest

The authors declare that the research was conducted in the absence of any commercial or financial relationships that could be construed as a potential conflict of interest.

## Publisher’s Note

All claims expressed in this article are solely those of the authors and do not necessarily represent those of their affiliated organizations, or those of the publisher, the editors and the reviewers. Any product that may be evaluated in this article, or claim that may be made by its manufacturer, is not guaranteed or endorsed by the publisher.
